# Regular meals matter: bone growth and beyond

**DOI:** 10.1172/JCI194079

**Published:** 2025-06-16

**Authors:** Rhonda D. Kineman, Shoshana Yakar

**Affiliations:** 1 Section of Endocrinology, Diabetes, and Metabolism, Department of Medicine, University of Illinois at Chicago, Chicago, Illinois, USA.; 2 Research and Development Division, Jesse Brown VA Medical Center, Chicago, Illinois, USA.; 3 Department of Molecular Pathobiology, David B. Kriser Dental Center, New York University College of Dentistry, New York, New York, USA.

## Abstract

The effect of food intake patterns on growth remain largely unknown. In this issue of the *JCI*, Hornsby et al. provide compelling evidence that, in young males, confining food intake to three meals a day entrains preprandial ghrelin release, leading to postprandial growth hormone pulse release that is associated with an increase in epiphysial plate expansion — a measure indicative of increased bone growth. The positive effects of discrete meal intake, on bone, was dependent on an intact ghrelin signaling system. This Commentary posits that meal-entrained ghrelin release may enhance skeletal accrual, whether through direct action on bone cells, via stimulation of growth hormone secretion, or in concert with other nutrient-responsive hormones. Coordinating these hormonal cues with food intake could maximize bone acquisition and improve bone health throughout the lifespan.

## Coordination of nutrients and growth hormone

**Figure 1:**
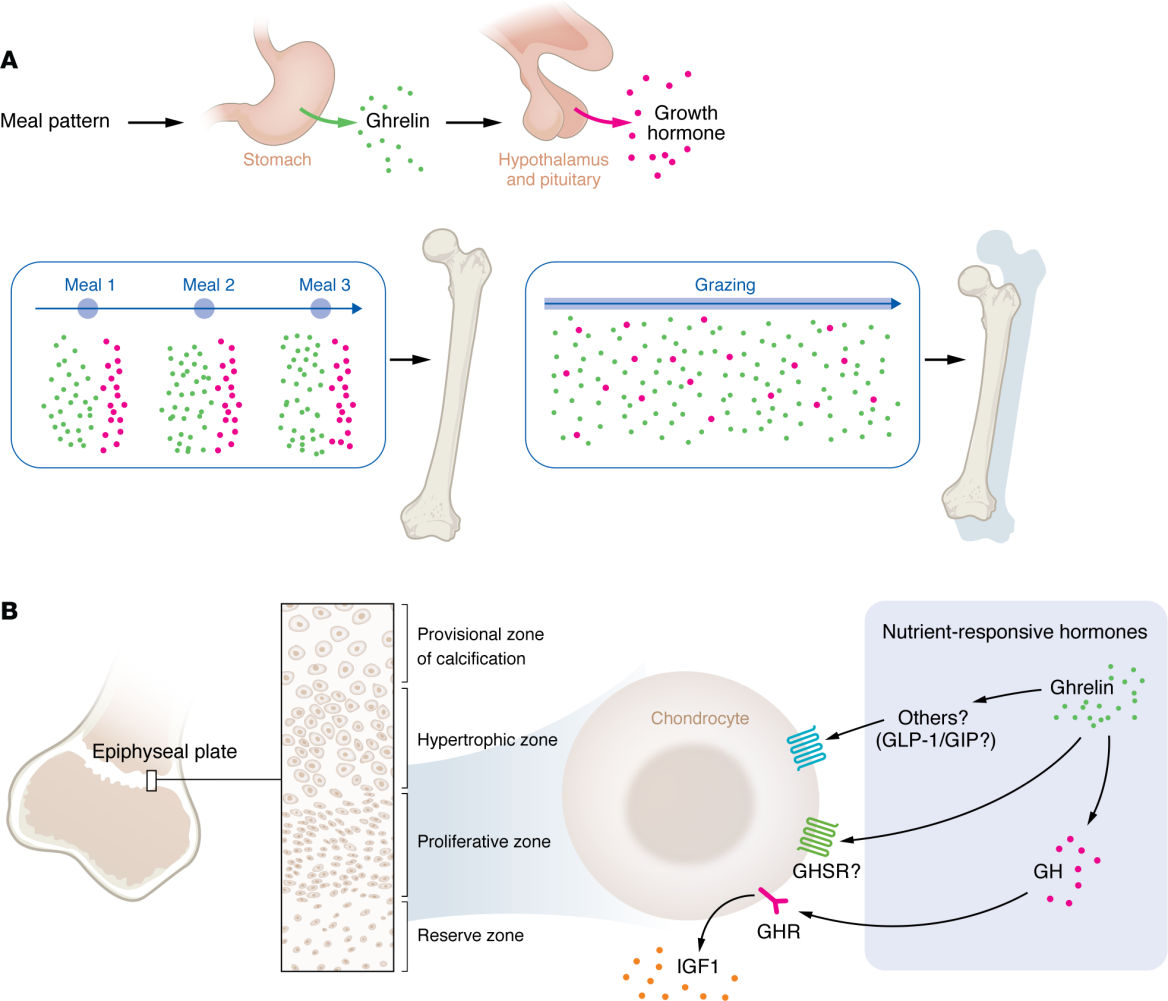
The pattern of food intake affects bone growth. (**A**) In male rats and human volunteers, food intake in a pattern of three meals a day entrained preprandial ghrelin release, leading to high-level pulses of postprandial GH. This pattern in rats resulted in epiphysial plate expansion, which is a hallmark of enhanced longitudinal growth. In contrast, a grazing pattern led to minimized ghrelin and GH rhythmicity and no changes in the epiphyseal plate width. (**B**) Meal-entrained ghrelin release may increase bone growth directly or indirectly via stimulation of GH secretion, or it could cooperate with other nutrient-responsive hormones to elongate bones. Whether pattern feeding induces endocrine rhythms that result in cyclic GH-dependent gene expression within chrondrocytes or other cells of the growth plate remains to be determined. GHR, GH receptor.

While the quality and quantity of nutrition are associated with health and growth, less is known about how the eating schedule affects development. In this issue of the *JCI*, Hornsby et al. (1) provide compelling cross-species evidence that the pattern of food intake also plays a significant role for bone growth beyond that provided by the amount of calories consumed. In male rats and mice, consolidation of food intake into three discrete meals (versus an isocaloric grazing pattern) widened the tibial epiphyseal plate, which is a hallmark of enhanced longitudinal growth. This skeletal outcome disappeared in ghrelin or ghrelin receptor–null mice, suggesting links among the feeding schedule, the ghrelin/growth hormone (ghrelin/GH) axis, and growth plate biology. Such a connection was further supported by the observation that, in rats, discrete meal feeding compared with grazing was associated with more dynamic ghrelin release and enhanced GH pulse pattern, without elevating circulating insulin-like growth factor 1 (IGF1) levels. Similarly, in humans, nasogastric delivery of bolus meals, but not continuous feeding, was associated with preprandial ghrelin peaks and ultradian GH rhythmicity, suggesting evolutionary conservation of this timing circuit (Figure 1).

Efficient partitioning of macronutrients toward somatic growth relies on tight hormonal coordination. During puberty, the growth plate operates at maximal velocity in the context of transient insulin resistance, which is partly driven by high GH levels (2). This hormonal environment promotes hepatic glucose output and adipose tissue lipolysis (3), ensuring an energy-rich milieu when resting zone chondrocytes enter the cell cycle. By concentrating intake into discrete meals, preprandial ghrelin release from the stomach entrains hypothalamic and pituitary regulation of GH pulse release (4) that would be predicted to act directly on chondrocytes to expand the growth plate (5, 6). Although Hornsby et al. (1) found meal feeding did not alter circulating IGF1 levels, it is important to note that cartilage and bone produce their own IGF1 in response to GH stimulation (7). Whether pattern feeding–induced, endocrine rhythms translate into oscillatory GH-dependent gene expression within the growth plate remains unanswered.

## Patterns of GH secretion

Understanding how endocrine rhythms may affect bone growth requires a brief return to the classic endocrine principle of desensitization, which occurs after prolonged hormone exposure. Consistent with this concept, intermittent, but high, GH pulses drive STAT5b signaling in target tissues far more effectively than does continuous delivery of the same amount of hormone over the same period. Importantly, the pattern of GH release differs between the sexes, pulsatile in males versus more continuous in females, which has been found to have a profound effect on liver function (8–10). Yet, no sex-specific longitudinal studies have correlated GH burst frequency or amplitude with bone mineral acquisition. Hornsby et al. (1) show that meal feeding dramatically increased GH secretory bursts in male rats and humans. However, it remains to be determined whether female GH release patterns and bone growth are also sensitive to meal patterns. Absolute caloric intake, as well as meal patterns modulate insulin and leptin, both key regulators of gonadotropin-releasing hormone (GnRH) and, downstream, gonadal steroidogenesis. Estradiol hastens growth plate fusion, whereas testosterone enhances periosteal expansion (11). Future work should dissect whether meal-induced ghrelin/GH rhythms alter the timing or magnitude of the pubertal sex steroid surge, thereby indirectly affecting bone geometry. The window of opportunity for optimizing peak bone mass may, therefore, be defined by the convergence of feeding habits, GH pulsatility, and sex steroid exposure. Given this complex interplay between hormonal signals and bone, the data presented by Hornsby et al. (1) invite a reexamination of whether today’s snack culture could blunt this synchrony and undermine peak bone mass accrual.

Adding another layer of complexity is the consideration for growth plate chondrocytes, which produce ghrelin and express its receptor, GHSR1a. In primary human articular chondrocytes, ghrelin transcripts are enriched, and exogenous ghrelin boosts SOX9 and type II collagen, hallmarks of chondrogenesis (12). In complementary in vitro models, ghrelin accelerates mesenchyme-to-chondrocyte differentiation through ERK1/2 signaling and protects the matrix from catabolic insults, highlighting a direct autocrine-paracrine route (13). In addition to the direct and GH-dependent actions of ghrelin, ghrelin also regulates the release and function of meal-induced incretins (glucagon-like peptide 1 [GLP-1] and gastric inhibitory polypeptide [GIP]) (14), gut peptides that are emerging as contributors to bone health and specifically affect the bone remodeling process (15). In addition, GLP-1 was found to enhance GH release (16). Collectively, these findings raise the intriguing possibility that the consolidated meal ghrelin surge described by Hornsby et al. (1) works at multiple levels to synchronize cues that promote chondrocyte proliferation and matrix synthesis.

## Skeletal acquisition

Even during growth, widening an epiphyseal plate is not synonymous with stronger bone. Epiphyseal plate width predicts longitudinal gain, but skeletal robustness equally depends on cortical thickness, trabecular microarchitecture, and matrix mineralization. Hornsby et al. (1) did not report geometric or material properties, leaving it to be determined whether meal patterns merely elongate bones or also enhance their strength, especially since GH excess can, paradoxically, reduce material density despite increasing size (17, 18).

These developmental considerations naturally raise the question of relevance after linear growth has ceased. Unlike rodents, humans lose their growth plates after puberty (19); however, periosteal apposition and remodeling continue throughout life. GH pulses persist in adulthood and decline with age, paralleling cortical porosity and fracture risk. Thus, temporal feeding might still modulate skeletal maintenance across lifespan via osteoblast-lineage cells, osteoclasts, and marrow adipocytes. Interventional trials in young adults, in whom height is stable, but modeling persists, could determine whether limiting food intake to discrete meals augments GH pulsatility and slows age-related bone loss. It is also tempting to speculate that meal-induced GH pulsatility in adults may also be beneficial to preserve overall metabolic health, since GH replacement in GH-deficient adults serves to improve body composition, prevent liver steatosis, and promote cardiometabolic health (20–22).

## Implications and conclusions

Translational implications come into sharp focus when we consider current GH replacement strategies. For over 30 years, pediatric and adult patients with GH deficiency (GHD) have been treated with single, daily GH injections recommended to be administered before sleep to better mimic the elevated nocturnal GH levels observed in GH-replete individuals. More recently, long-acting GH preparations have been FDA approved, with the same safety profile and improved compliance. However, it remains to be seen whether, with similar compliance, the long-acting formulation matches the effectiveness of the single-injection forms in all key outcomes, including bone growth and health (23). We may speculate that the long-acting formulations may blunt anabolic signaling, mimicking the continuous-feed paradigm that flattens GH rhythms in humans. However, it may be possible to combine GH therapy with regular meal intake to improve outcomes, particularly the related bone health as reviewed above.

In summary, Hornsby et al. (1) elegantly demonstrate that the skeleton is not blind to the clock on the kitchen wall. By stitching together meal-entrained ghrelin surges with amplified GH pulses, the authors reveal a temporal endocrine code that optimizes chondrocyte proliferation and linear growth (Figure 1). The findings resonate with broader concerns that 24-hour grazing, a hallmark of modern lifestyles, may compromise not only metabolic health but also skeletal potential. Dissecting how feeding schedules intersect with sex steroids, local IGF1, and bone quality determinants promises a fertile field for both basic and translational research. As we refine guidelines for pediatric nutrition and GH therapeutics, when we eat may prove just as critical for building strong bones as what and how much we eat.

## Version 1 06/16/2025

Electronic publication
